# Treatment discontinuation following low-dose TKIs in 248 chronic myeloid leukemia patients: Updated results from a campus CML real-life study

**DOI:** 10.3389/fphar.2023.1154377

**Published:** 2023-03-23

**Authors:** A. Iurlo, D. Cattaneo, D. Consonni, F. Castagnetti, M. C. Miggiano, G. Binotto, M. Bonifacio, G. Rege-Cambrin, M. Tiribelli, F. Lunghi, A. Gozzini, P. Pregno, E. Abruzzese, I. Capodanno, C. Bucelli, M. Pizzuti, S. Artuso, M. Iezza, E. Scalzulli, G. La Barba, A. Maggi, S. Russo, C. Elena, A. R. Scortechini, A. Tafuri, R. Latagliata, G. Caocci, M. Bocchia, S. Galimberti, L. Luciano, C. Fava, R. Foà, G. Saglio, G. Rosti, M. Breccia

**Affiliations:** ^1^ Hematology Division, Foundation IRCCS Ca’ Granda Ospedale Maggiore Policlinico, Milan, Italy; ^2^ Department of Oncology and Hemato-Oncology, University of Milan, Milan, Italy; ^3^ Epidemiology Unit, Foundation IRCCS Ca’ Granda Ospedale Maggiore Policlinico, Milan, Italy; ^4^ Department of Experimental, Diagnostic and Specialty Medicine, Institute of Hematology “L. and A. Seràgnoli”, University of Bologna, “S. Orsola-Malpighi” Hospital, Bologna, Italy; ^5^ Division of Hematology, San Bortolo Hospital, Vicenza, Italy; ^6^ Department of Medicine, Hematology and Clinical Immunology, Padua School of Medicine, Padua, Italy; ^7^ Department of Medicine, Section of Hematology, University of Verona, Verona, Italy; ^8^ Division of Internal Medicine and Hematology, San Luigi Gonzaga Hospital, Turin, Italy; ^9^ Division of Hematology and BMT—Udine Hopsital, ASUFC and Department of Medicine—University of Udine, Udine, Italy; ^10^ Division of Hematology and BMT, IRCCS San Raffaele Hospital, Milan, Italy; ^11^ Division of Hematology, AOU Careggi, Firenze, Italy; ^12^ Division of Hematology, AOU Città della Salute e della Scienza, Torino, Italy; ^13^ Hematology Division, Sant’Eugenio Hospital, Rome, Italy; ^14^ Division of Hematology, IRCCS Arcispedale Santa Maria Nuova, Reggio Emilia, Italy; ^15^ Hematology Unit, Ospedale Potenza, Potenza, Italy; ^16^ Division of Hematology, Department of Precision and Translational, Policlinico Umberto 1, Sapienza University, Rome, Italy; ^17^ Hematology Unit, Azienda USL di Pescara, Pescara, Italy; ^18^ Division of Hematology, Hospital “S. G. Moscati”, Taranto, Italy; ^19^ Division of Hematology, Dipartimento di Patologia Umana dell''Adulto e dell'Età Evolutiva, Policlinico G. Martino, University of Messina, Messina, Italy; ^20^ UOC Ematologia 1, Fondazione IRCCS Policlinico San Matteo, Pavia, Italy; ^21^ Division of Hematology, Department of Molecular and Clinical Sciences, Polytechnic University of Marche, Ancona, Italy; ^22^ Division of Hematology, Azienda Ospedaliera Universitaria Sant'Andrea, Rome, Italy; ^23^ Division of Hematology, Belcolle Hospital, Viterbo, Italy; ^24^ Department of Medical Sciences and Public Health, University of Cagliari, Businco Hospital, Cagliari, Italy; ^25^ Hematology Unit, Azienda Ospedaliera Universitaria Senese, University of Siena, Siena, Italy; ^26^ Department of Clinical and Experimental Medicine, University of Pisa, Pisa, Italy; ^27^ Division of Hematology, Department of Clinical Medicine and Surgery, Federico II University, Napoli, Italy; ^28^ Department of Clinical and Biological Sciences, University of Torino, Torino, Italy; ^29^ Scientific Direction, IRCCS Istituto Romagnolo per lo Studio dei Tumori (IRST) "Dino Amadori", Meldola, Italy

**Keywords:** chronic myeloid leukemia, tyrosine kinase inhibitors, treatment-free remission, low dose, resistance, outcome

## Abstract

TKIs long-term treatment in CML may lead to persistent adverse events (AEs) that can promote relevant morbidity and mortality. Consequently, TKIs dose reduction is often used to prevent AEs. However, data on its impact on successful treatment-free remission (TFR) are quite scarce. We conducted a retrospective study on the outcome of CML subjects who discontinued low-dose TKIs from 54 Italian hematology centers participating in the Campus CML network. Overall, 1.785 of 5.108 (35.0%) regularly followed CML patients were treated with low-dose TKIs, more frequently due to relevant comorbidities or AEs (1.288, 72.2%). TFR was attempted in 248 (13.9%) subjects, all but three while in deep molecular response (DMR). After a median follow-up of 24.9 months, 172 (69.4%) patients were still in TFR. TFR outcome was not influenced by gender, Sokal/ELTS risk scores, prior interferon, number and last type of TKI used prior to treatment cessation, DMR degree, reason for dose reduction or median TKIs duration. Conversely, TFR probability was significantly better in the absence of resistance to any prior TKI. In addition, patients with a longer DMR duration before TKI discontinuation (i.e., >6.8 years) and those with an e14a2 *BCR::ABL1* transcript type showed a trend towards prolonged TFR. It should also be emphasized that only 30.6% of our cases suffered from molecular relapse, less than reported during full-dose TKI treatment. The use of low-dose TKIs does not appear to affect the likelihood of achieving a DMR and thus trying a treatment withdrawal, but might even promote the TFR rate.

## 1 Introduction

The therapeutic armamentarium of chronic myeloid leukemia (CML) has greatly improved after small molecule tyrosine kinase inhibitors (TKIs) targeting *BCR::ABL1* became available. The 10-year survival rate in CML-chronic phase increased from about 20% to 80%–90%, allowing for near-normal life expectancy (Bower et al., 2016).

While highly effective, these drugs also have an often mild to moderate, but sometimes severe, toxicity profile. Indeed, long-term treatment with TKIs may lead to chronic adverse events (AEs) that can negatively affect patients’ quality of life (QoL) and can promote relevant morbidity and mortality. In particular, there has been increasing evidence of more serious AEs with second- or third-generation TKIs, such as pleural effusion and pulmonary arterial hypertension with dasatinib (Campbell and Copland, 2013), hyperglycemia, dyslipidemia and arterial occlusive events (AOEs) with nilotinib (Emir et al., 2013; Hiwase et al., 2013), gastrointestinal toxicities with bosutinib (Cortes et al., 2012), and hypertension, AOEs and pancreatic dysfunction with ponatinib (Cortes et al., 2018).

Dose reductions due to AEs are part of the daily management of TKIs (Copland, 2019): often these dose reductions are kept stable over the long term, particularly in patients who have already achieved a durable response. This optimization of the TKI dose is related to better adherence, improved QoL and in most cases do not jeopardize a stable response once achieved, thus confirming, as studies on intermittent TKI treatment have already shown (Russo et al., 2013; Malagola et al., 2021), that responding patients are often overtreated. Furthermore, as also demonstrated by the UK DESTINY study (Clark et al., 2017; Clark et al., 2019) and real-world data from Hammersmith Hospital (Claudiani et al., 2021), a TKI de-escalation strategy, in addition to not compromising the possibility of treatment discontinuation, could also improve the success of treatment-free remission (TFR) protocols.

However, while discontinuing therapy appears as a safe option for approximately half of the patients obtaining an optimal response (Ross and Hughes, 2020), so far, data on the impact of long-term TKI dose de-escalation on successful TFR are rather scarce.

The aim of our study was therefore to evaluate the propensity of Italian hematologists to attempt TFR in CML patients treated with low-dose TKIs, both at diagnosis and during follow-up.

## 2 Methods

We conducted a retrospective analysis on the outcome of CML patients who discontinued low-dose TKIs between May 2010 and September 2022 from 54 Italian hematology centers participating to the “Campus CML”, an active research network of physicians involved in CML management throughout Italy, with the aim of investigating different aspects of the disease (Iurlo et al., 2022).

Each center was asked to complete an online survey which included questions regarding the use of low-dose TKIs in real-life clinical practice and the willingness of physicians to offer TFR even to subjects treated with reduced doses of TKI for AEs and/or relevant comorbidities or to patients who have already achieved an optimal molecular response.

The following key disease characteristics were then required for each patient attempting TFR after low-dose TKI treatment: socio-demographic variables, risk scores (i.e., Sokal and ELTS), all treatments (including interferon) before and after discontinuation, duration of each treatment, reasons for dose reduction, and best response to each treatment.

Molecular monitoring and classification of responses were defined according to current ELN recommendations (Hochhaus et al., 2020): in particular, major molecular response (MMR) as a *BCR::ABL/ABL* ratio ≤ 0.1%, and deep molecular response (DMR) as MR4 (*BCR::ABL1/ABL* ratio ≤ 0.01%), MR4.5 (*BCR::ABL1/ABL* ratio ≤ 0.0032%), or MR5 (*BCR::ABL1/ABL* ratio ≤ 0.001%). Molecular relapse was defined as loss of MMR. Consequently, TFR was calculated from TKI discontinuation to loss of MMR.

Follow-up began on the day of TKI discontinuation and ended at the earliest of molecular relapse or 31 December 2022. As only 65 (26.2%) patients were followed up for more than 4 years and no molecular recurrence was observed among them, we truncated follow-up time at 4 years.

We analyzed time since to molecular relapse based on selected variables using the Kaplan-Meier survivor function and the log-rank-test. Statistical analyses were performed with Stata 17 (StataCorp. 2021).

## 3 Results

Overall, 1.785 of 5.108 (35.0%) regularly followed CML-chronic phase patients were treated with low-dose TKIs. More specifically, a TKI dose reduction was reported in 727 (27.5%) out of 2.648 patients treated with imatinib, 362/1.133 (32.0%) with nilotinib, 304/813 (37.4%) with dasatinib, 213/294 (72.5%) with bosutinib and 179/220 (81.4%) with ponatinib. The TKI dose was reduced in the majority of patients (1.288, 72.2%) due to AEs (916, 51.3%) or relevant comorbidities (372, 20.8%), while in the remaining subjects (497, 27.8%) TKIs were reduced for physicians’ decision, after obtaining a stable molecular response (MMR/DMR), with the aim of preventing the development of AEs, thereby improving TKIs tolerability. No progression toward advanced-phase disease was observed during treatment with low-dose TKIs.

TFR was attempted in 248 (13.9%) out of 1.785 patients, 138 of whom were female and 110 were male, with a median age at CML diagnosis of 49.3 years (Table 1). As expected, dose reduction was due to AEs in more than half of the cases (149 patients, 60.1%), while in the remaining subjects TKIs were reduced after reaching an optimal molecular response (Supplementary Table S1). Interestingly, 47 (18.9%) patients experienced TKI resistance before treatment discontinuation, in the absence of any *BCR::ABL1* kinase domain mutation.

**TABLE 1:** Baseline demographics and clinical characteristics of 248 CML patients.

Characteristics	Patients (N = 248)
M/F	110/138
Age at CML diagnosis (years), median (range)	49.3 (19.1–81.4)
Sokal risk, n (%)	
Low	122 (49.2)
Intermediate	78 (31.5)
High	33 (13.3)
NA	15 (6.0)
ELTS risk, n (%)	
Low	181 (73.0)
Intermediate	37 (14.9)
High	13 (5.2)
NA	17 (6.9)
*BCR::ABL1* p210 transcript type, n (%)	
e14a2	155 (62.5)
e13a2	56 (22.6)
e14a2/e13a2	19 (7.7)
Other types	8 (3.2)
NA	10 (4.0)
Previous IFN, n (%)	39 (15.7)
Time from diagnosis to TKI discontinuation (months), median (range)	115.2 (16.0–355.9)
Duration of TKI therapy (months), median (range)	111.2 (16.0–252.1)
Duration of low-dose TKI (months), median (range)	37.4 (0.6–197.9)
Age at TKI discontinuation (years), median (range)	61.9 (29.7–89.2)
MR at TKI discontinuation, n (%)	
MMR	3 (1.2)
MR4.0	55 (22.2)
MR4.5	96 (38.7)
MR5.0	94 (37.9)
Duration of DMR before TKI discontinuation (months), median (range)	65.0 (3.0–186.0)

Abbreviations: CML, chronic myeloid leukemia; NA, not available; MR, molecular response; MMR, major molecular response; DMR, deep molecular response.

The most widely used low-dose TKI before attempting TFR was imatinib (99 patients out of 727–13.6%), followed by nilotinib (90 patients out of 362–24.9%), dasatinib (51 patients out of 304–16.8%) and, to a lesser extent, ponatinib (five patients out 179–2.8%) and bosutinib (three patients out of 213–1.4%). Overall, 117 (47.2%) patients experienced a ≥ 50% reduction in TKI dose from baseline (Supplementary Table S2).

Before attempting TFR, 152 (61.3%) patients were treated with a single TKI, while 80 and 16 patients had received second or later-line therapy, respectively, with a median TKI treatment duration of 111.2 months (range, 16.0–252.1). At the time of TFR, 245 (98.8%) patients had already achieved a DMR, with a median duration of 65.0 months (range, 3.0–186.0).

**FIGURE 1:**
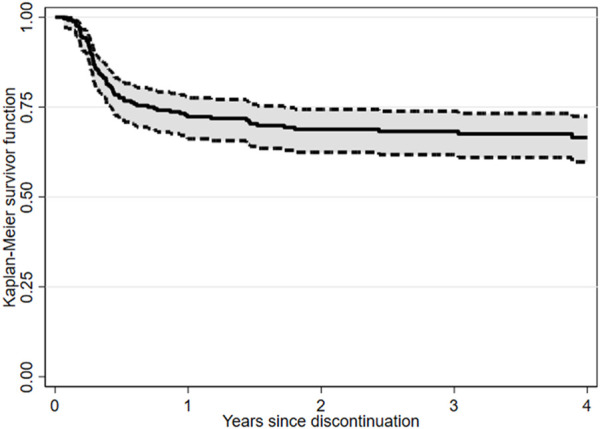
Treatment-free remission (TFR) after tyrosine kinase inhibitor (TKI) therapy (Kaplan-Meier survivor function).

**FIGURE 2:**
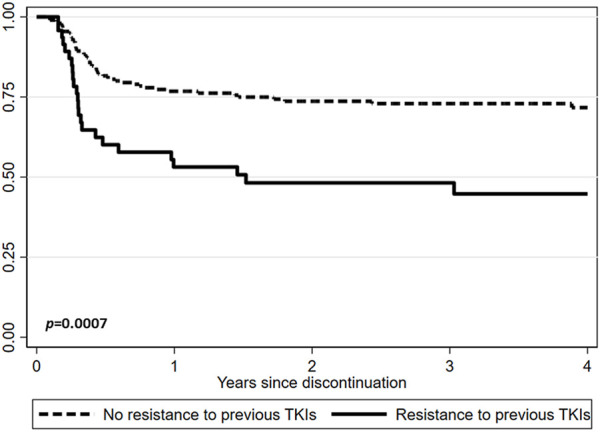
Treatment-free remission (TFR) after tyrosine kinase inhibitor (TKI) therapy (Kaplan-Meier survivor function) according to resistance to previous TKIs.

After a median follow-up from TKIs discontinuation of 24.9 months (range, 1.1–149.2), 172 (69.4%) patients were still in TFR (Figure 1). Interestingly, TFR outcome was not affected by any of the following parameters (Supplementary Table S3): gender, Sokal or ELTS risk scores, prior interferon treatment, number and last type of TKIs used before discontinuation of therapy, degree of DMR (i.e., MR4 vs. MR4.5 or better), reason for dose reduction (i.e., AEs vs. DMR achievement) or median duration of therapy with TKIs (Supplementary Figures S1, S2). In contrast, TFR was markedly better in the absence of a history of resistance to any previous TKI (Table 2; Figure 2); furthermore, as expected, patients with a longer DMR duration (i.e., >6.8 years) (Supplementary Figure S2) and those with an e14a2 *BCR::ABL1* transcript type showed a trend toward prolonged TFR (Table 2).

**TABLE 2:** Molecular recurrence risk and hazard ratios from univariate Cox models according to selected variables.

	N. patients	N. molecular recurrence (%)	HRs	95% CI	*p*
Gender					
M	110	30 (27.3)	1.00	Reference	
F	138	46 (33.3)	1.28	0.81–2.02	0.30
Sokal risk					
Low	122	39 (32.0)	1.00	Reference	
Intermediate	78	20 (25.6)	0.81	0.47–1.38	0.43
High	33	15 (45.4)	1.55	0.86–2.82	0.15
ELTS risk					
Low	181	58 (32.0)	1.00	Reference	
Intermediate	37	13 (35.1)	1.11	0.61–2.03	0.73
High	13	3 (23.1)	0.73	0.23–2.34	0.60
Transcript type					
e14a2	155	44 (28.4)	1.00	Reference	
e13a2	56	22 (39.3)	1.65	0.99–2.75	**0.06**
e14a2/e13a2	19	7 (36.8)	1.36	0.61–3.03	0.44
Previous IFN					
NO	209	67 (32.1)	1.00	Reference	
YES	39	9 (23.1)	0.62	0.31–1.24	0.18
First-line TKI					
Imatinib	177	54 (30.5)	1.00	Reference	
Dasatinib	20	10 (50.0)	1.73	0.88–3.41	0.11
Nilotinib	51	12 (23.5)	0.81	0.43–1.51	0.50
Reason for dose reduction					
MR achievement	100	32 (32.0)	1.00	Reference	
AEs	148	44 (29.7)	0.87	0.55–1.37	0.55
Duration of TKIs (years)					
<10	142	45 (31.7)	1.00	Reference	
≥10	106	31 (29.2)	0.89	0.56–1.40	0.60
Last TKI at discontinuation					
Imatinib	99	27 (27.3)	0.75	0.47–1.21	0.24
2/3G-TKIs	149	49 (32.9)	1.00	Reference	
Line of therapy at discontinuation					
First-line	152	45 (29.6)	0.82	0.52–1.30	0.41
Second- or later lines	96	31 (32.3)	1.00	Reference	
Resistance to previous TKIs					
YES	47	24 (51.1)	1.00	Reference	
NO	201	52 (25.9)	2.45	1.51–3.98	**0.000**
DMR degree at TFR					
MR4.0	55	18 (32.7)	1.00	Reference	
≥MR4.5	190	56 (29.5)	0.86	0.50–1.46	0.57
Duration of DMR (years)					
<4.2	84	32 (38.1)	1.00	Reference	
4.2–6.7	81	24 (29.6)	0.71	0.42–1.21	0.21
≥6.8	83	20 (24.1)	0.57	0.33–0.99	**0.05**

Abbreviations: M, male; F, female; MR, molecular response; AEs, adverse events; DMR, deep molecular response; HR, hazard ratio; CI, confidence interval. Bold values are statistically significant.

Seventy-six (30.6%) patients experienced molecular recurrence (≤MMR) after a median time from treatment interruption of 4.1 months (range, 0.8–47.3), representing an early relapse (within 6 months) in 54/76 patients (71.1%). All of these cases who had a molecular relapse restarted the same dose of TKI treatments they had before the TFR attempt and regained at least an MMR within 8 months of TKI resumption.

## 4 Discussion

In this large, real-life series of CML patients regularly followed in Italy, TKIs treatment at a reduced dose represents an important reality, being bosutinib and ponatinib the drugs that most frequently required a dose reduction. More notably, TKI treatment has usually been initiated at full dose (excluding the elderly or patients with relevant comorbidities), with nearly 30% of TKI dose reductions performed in clinical practice with the goal of long-term treatment improvement once an optimal molecular response has been obtained, while at the same time preventing the development of AEs, thus improving TKIs tolerability.

Indeed, it should be emphasized that TKI dose optimization should be taken into account from the outset as, after the development of chronic toxicities, the true possibility of complete resolution of AEs is still a matter of debate, especially in some specific contexts (Okamoto et al., 2020).

In fact, while all the information on the prescription of TKIs used in CML treatment indicate a starting dose and rules and suggestions for transiently suspending or reducing the dose in case of clinical and biochemical AEs, suggesting a return to the ideal dose once the AEs disappear or alleviate, in daily clinical practice many physicians prescribe TKI starting dose lower than those foreseen in the product information in case of patients of advanced age or with relevant comorbidities. Furthermore, when an AEs improves or disappears, the TKI is maintained at a reduced dose over the long term, essentially to avoid recurrence of the same AE or, generically, to promote a better QoL and to ensure or reassure a good adherence, thus allowing a broader use of even those second- or third-generation TKIs not adequately indicated in the presence of specific comorbidities (Iurlo et al., 2021).

Furthermore, while most studies of TFR in CML subjects abruptly discontinued TKIs, more recently some authors have evaluated a new treatment strategy based on gradual therapy withdrawal before TKI interruption in the context of clinical trials or real-life experiences (Clark et al., 2017; Clark et al., 2019; Cayssials et al., 2020; Claudiani et al., 2021): among others, the DESTINY study examined TKI de-escalation treatment in 174 patients with stable MMR or MR4, after at least 3 years of treatment with full-dose imatinib, nilotinib, or dasatinib (Clark et al., 2017). There was no progression or cytogenetic recurrence, with monthly monitoring allowing rapid identification of all cases of molecular relapse. Furthermore, chronic AEs improved in most cases (Clark et al., 2019).

More recently, the prospective, multicenter phase II DANTE study (NCT03874858) aimed to evaluate the safety of first-line nilotinib de-escalation and its impact on TFR success in Italian CML-chronic phase subjects. While in a previously interim analysis 1 year of nilotinib de-escalation prior to TFR in patients with stable DMR was shown to be safe and effective (Breccia et al., 2021), a molecular recurrence rate of approximately 32% 1 year after stopping nilotinib was reported, thus demonstrating that de-escalation of this drug before attempting TFR may be a successful dose optimization strategy (Stagno et al., 2022).

Accordingly, we then evaluated the potential effect of low-dose TKIs on TFR outcome in a large real-life series of 248 CML-chronic phase patients managed in Italy.

In this context, as already reported in a previous paper (Iurlo et al., 2022), we confirm in a larger patients’ population that the use of low-dose TKIs does not seem to affect the possibility of achieving a DMR and therefore attempting a suspension of therapy.

While being aware of the limitations of this study, mainly represented by its retrospective nature, it should be emphasized that only 30.6% of our cases suffered from molecular relapse, less than reported in patients previously treated with full-dose TKIs (Hernández-Boluda et al., 2018; Fava et al., 2019).

In addition, we have now demonstrated that the only clinical variable associated with molecular recurrence was resistance to prior TKIs (Figure 2), thus confirming observations from previous studies, for example, the DADI trial in which patients who switched to dasatinib because of resistance to imatinib had worse outcomes than those who switched for other reasons (Imagawa et al., 2015). Similarly, in the more recent STOP 2G-TKI French study a history of suboptimal response or resistance to imatinib prior to dasatinib or nilotinib was the only baseline factor associated with significantly worse TFR (Rea et al., 2017). Consequently, it must be admitted that in this context only a select subset of patients has the possibility of achieving a successful TFR, probably for a less aggressive disease.

In fact, approximately 14.0% of our patients treated with TKIs at a reduced dose were considered eligible to discontinue therapy, thus demonstrating that TFR was not compromised using low-dose TKIs. Furthermore, treatment dose adjustments have important prognostic implications even in those patients who do not wish to attempt TFR, with the clinical potential to reduce both therapy-related AEs and overall treatment costs without jeopardizing the possibility of maintaining a good response.

## Data Availability

The original contributions presented in the study are included in the article/Supplementary Material, further inquiries can be directed to the corresponding author.

## Supplementary material

SUPPLEMENTARY FIGURE S1 Treatment-free remission (TFR) after tyrosine kinase inhibitor (TKI) therapy (Kaplan-Meier survivor function). **(A)** TFR according to gender. **(B)** TFR according to pretreatment with interferon. **(C)** TFR according to Sokal risk score. **(D)** TFR according to TKIs used before therapy cessation.

SUPPLEMENTARY FIGURE S2 Treatment-free remission (TFR) after tyrosine kinase inhibitor (TKI) therapy (Kaplan-Meier survivor function). **(A)** TFR according to *BCR::ABL1* transcript type. **(B)** TFR according to lines of therapy. **(C)** TFR according to median duration of TKIs therapy. **(D)** TFR according to median duration of DMR. **(E)** TFR according to the degree of DMR. **(F)** TFR according to the main reason for TKIs dose reduction.
