# Supplementary material for: Treatment discontinuation following low-dose TKIs in 248 chronic myeloid leukemia patients: Updated results from a campus CML real-life study
Source: Front Pharmacol. 2023 Mar 23;14:1154377. doi: 10.3389/fphar.2023.1154377 (PMC10076530; doi:10.3389/fphar.2023.1154377)
Supplement: Supplementary file 1 [file Table1.docx]

**Supplementary Table 1. Reasons for dose reduction according to each TKI.**

|  | **First-line (N = 152)** | **Second-line (N = 80)** | **Third- or later lines (N = 16)** | **Overall (N = 248)** |
| --- | --- | --- | --- | --- |
| **Imatinib**, n (AEs/MR) | 56/37 | 2/3 | 1/- | 59/40 |
| **Nilotinib**, n (AEs/MR) | 21/22 | 23/17 | 5/2 | 49/41 |
| **Dasatinib**, n (AEs/MR) | 12/4 | 19/12 | 4/- | 35/16 |
| **Bosutinib**, n (AEs/MR) | -/- | 1/- | 2/- | 3/- |
| **Ponatinib**, n (AEs/MR) | -/- | 1/2 | 2/- | 3/2 |
| **Overall**, n (AEs/MR) | 89/63 | 46/34 | 14/2 | 149/99 |

**Abbreviations:** AEs, adverse events; MR, molecular response.
