# Supplementary figures and images for: Treatment discontinuation following low-dose TKIs in 248 chronic myeloid leukemia patients: Updated results from a campus CML real-life study
Source: Front Pharmacol. 2023 Mar 23;14:1154377. doi: 10.3389/fphar.2023.1154377 (PMC10076530; doi:10.3389/fphar.2023.1154377)

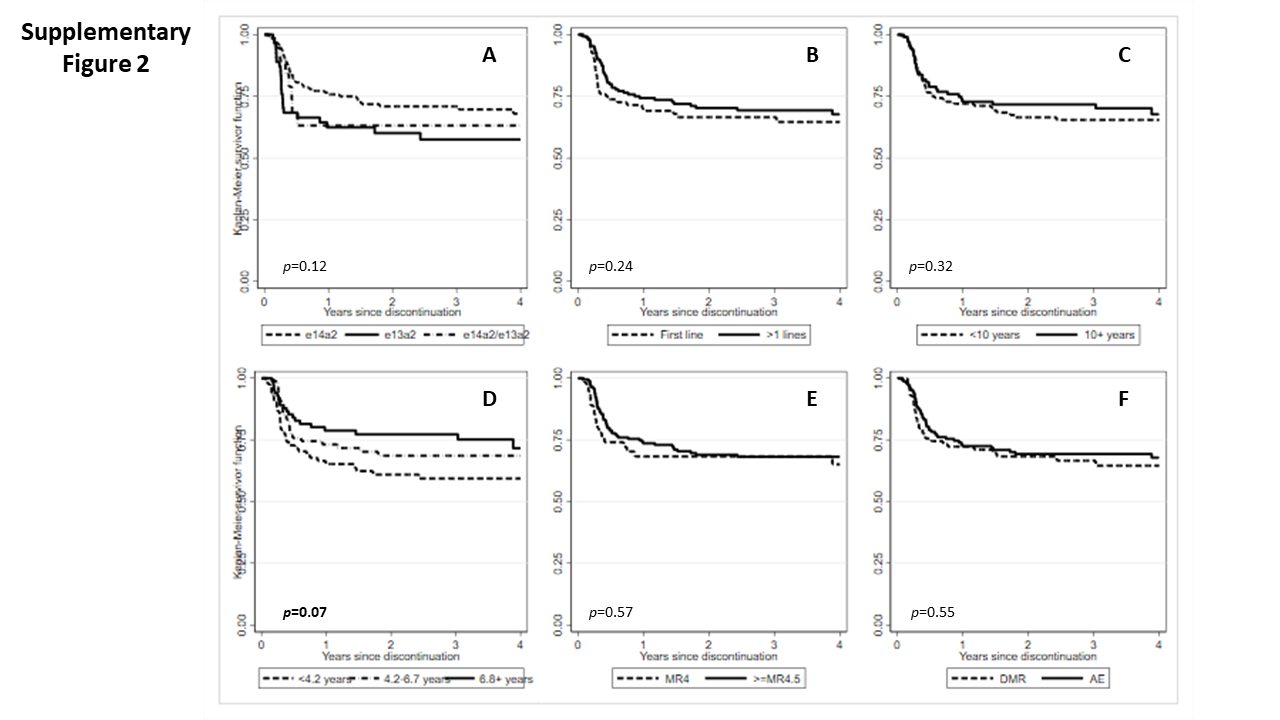

Supplement: Supplementary file 2 [file Image2.TIF]

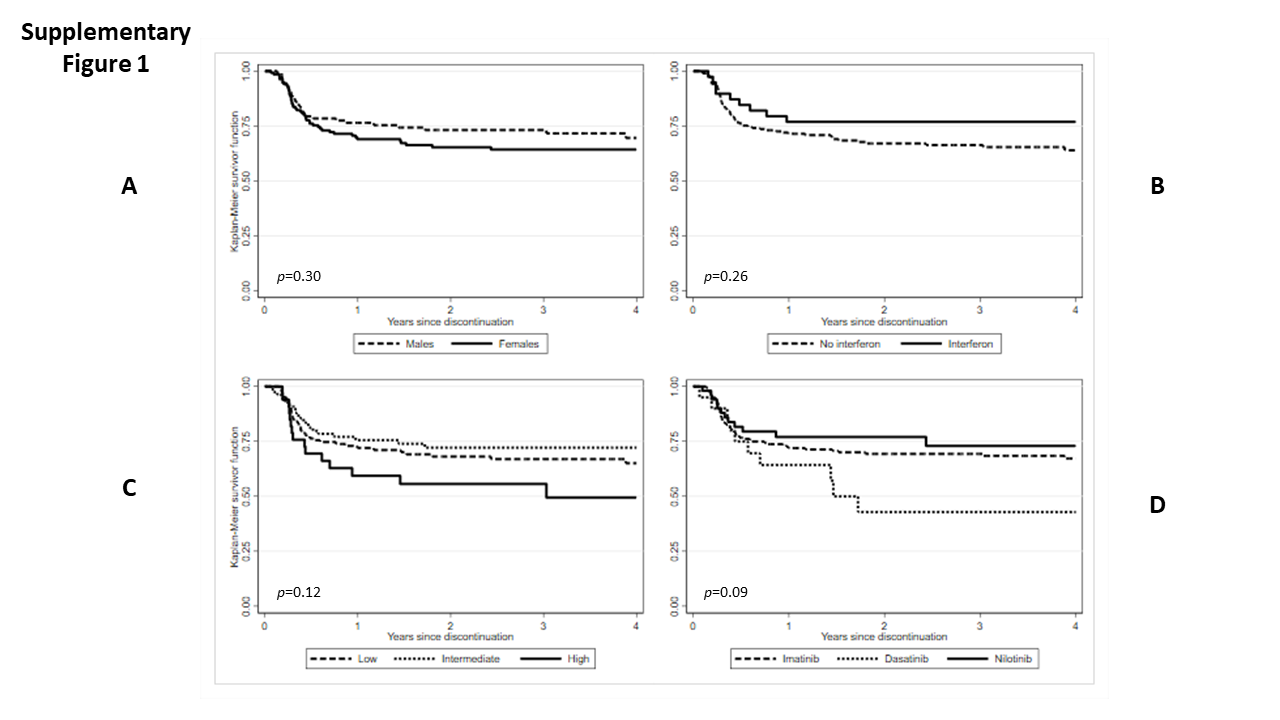

Supplement: Supplementary file 3 [file Image1.TIF]
