# Supplementary material for: Treatment discontinuation following low-dose TKIs in 248 chronic myeloid leukemia patients: Updated results from a campus CML real-life study
Source: Front Pharmacol. 2023 Mar 23;14:1154377. doi: 10.3389/fphar.2023.1154377 (PMC10076530; doi:10.3389/fphar.2023.1154377)
Supplement: Supplementary file 4 [file Table2.DOCX]

**Supplementary Table 2. Treatment characteristics.**

|  | **Patients (N = 248)** |
| --- | --- |
| **Last medication at TKI discontinuation**, n (%)  Imatinib  Nilotinib  Dasatinib  Bosutinib  Ponatinib | 99 (39.9)  90 (36.3)  51 (20.6)  3 (1.2)  5 (2.0) |
| **Dosage of imatinib before treatment cessation**, n (%)  200 mg/d  300 mg/d | 25 (25.3)  74 (74.7) |
| **Dosage of nilotinib before treatment cessation**, n (%)  200 mg/d  300 mg/d  400 mg/d  450 mg/d  600 mg/d | 6 (6.7)  24 (26.7)  27 (30.0)  19 (21.1)  14 (15.5) |
| **Dosage of dasatinib before treatment cessation**, n (%)  20 mg/d  50 mg/d  80 mg/d | 6 (11.8)  23 (45.1)  22 (43.1) |
| **Dosage of bosutinib before treatment cessation**, n (%)  200 mg/d  400 mg/d | 1 (33.3)  2 (66.7) |
| **Dosage of ponatinib before treatment cessation**, n (%)  15 mg/d | 5 (100.0) |
