# Supplementary material for: Treatment discontinuation following low-dose TKIs in 248 chronic myeloid leukemia patients: Updated results from a campus CML real-life study
Source: Front Pharmacol. 2023 Mar 23;14:1154377. doi: 10.3389/fphar.2023.1154377 (PMC10076530; doi:10.3389/fphar.2023.1154377)
Supplement: Supplementary file 5 [file Table3.docx]

**Supplementary Table 3. Univariate analysis of various parameters’ associations with treatment-free remission.**

| **Characteristic** | **Probability of maintaining TFR (%)** | ***p* (log-rank)** |
| --- | --- | --- |
| **Gender**  **M (N. 110)**  **F (N. 138)** | 72.3  66.7 | 0.30 |
| **Sokal risk**  **Low (N. 122)**  **Intermediate (N. 78)**  **High (N. 33)**  **NA (N. 15)** | 68.0  74.4  54.6  86.7 | 0.12 |
| **ELTS risk**  **Low (N. 181)**  **Intermediate (N. 37)**  **High (N. 13)**  **NA (N. 17)** | 68.0  64.9  76.9  88.2 | 0.73 |
| ***BCR::ABL1* p210 transcript type**  **e14a2 (N. 155)**  **e13a2 (N. 56)**  **e14a2/e13a2 (N. 19)** | 71.6  60.7  63.2 | 0.12 |
| **Previous IFN treatment**  **YES (N. 39)**  **NO (N. 209)** | 76.9  67.9 | 0.26 |
| **First-line TKI**  **Imatinib (N. 177)**  **Dasatinib (N. 20)**  **Nilotinib (N. 51)** | 69.5  50.0  76.5 | **0.09** |
| **Second-line TKI**  **Imatinib (N. 6)**  **Dasatinib (N. 38)**  **Nilotinib (N. 48)**  **Bosutinib (N. 1)**  **Ponatinib (N. 3)** | 66.7  65.8  66.7  100.0  100.0 | 0.74 |
| **Third-line TKI**  **Imatinib (N. 1)**  **Dasatinib (N. 5)**  **Nilotinib (N. 8)**  **Bosutinib (N. 2)** | 0.0  60.0  62.5  100.0 | 0.41 |
| **Reason for dose reduction**  **First-line TKI**  **AEs (N. 120)**  **MR (N. 65)**  **Second-line TKI**  **AEs (N. 54)**  **MR (N. 34)**  **Third-line TKI**  **AEs (N. 13)**  **MR (N. 2)** | 70.0  72.3  77.8  58.8  76.9  0.0 | 0.74  **0.06**  **0.03** |
| **Duration of TKIs (years)**  **<10 (N. 142)**  **>10 (N. 106)** | 68.3  70.8 | 0.32 |
| **Last TKI at discontinuation**  **Imatinib (N. 99)**  **2/3G-TKIs (N. 149)** | 72.7  67.1 | 0.24 |
| **Line of therapy at discontinuation**  **First-line (N. 152)**  **Second- or later lines (N. 96)** | 70.4  67.7 | 0.41 |
| **Resistance to previous TKIs**  **YES (N. 47)**  **NO (N. 201)** | 48.9  74.1 | **0.0007** |
| **MR at TKI discontinuation**  **MMR (N. 3)**  **MR4.0 (N. 55)**  **>MR4.5 (N. 190)** | 33.3  67.3  70.5 | 0.36 |
| **Duration of DMR (years)**  **<4.2 (N. 84)**  **4.2-6.7 (N. 81)**  **>6.8 (N. 83)** | 61.9  70.4  75.9 | **0.07** |

**Abbreviations:** TFR, treatment-free remission; M, male; F, female; NA, not available; MR, molecular response; DMR, deep molecular response; AEs, adverse events.
